# Using CK-MB and hs-cTnT to Predict 30-Day Mortality After CABG: A Combined Biomarker Risk-Stratification Approach

**DOI:** 10.1016/j.jacadv.2026.103284

**Published:** 2026-09-25

**Authors:** Winter-Pölzl Leo, Kletzer Joseph, Lohmann Ronja, Engler Clemens, Nägele Felix, Graber Michael, Hirsch Jakob, Heuts Samuel, J. Reinstadler Sebastian, Chaabi Zineb, Molander Linnea, Hammarsten Ola, Heitmann Leon Arnar, Czerny Martin, Holfeld Johannes, Griesmacher Andrea, Hartikainen Tau, Kreibich Maximilian, Grimm Michael, Bonaros Nikolaos, Berger Tim, Gudbjartsson Tomas, Jeppsson Anders, Willeit Peter, Gollmann-Tepeköylü Can

**Affiliations:** a Department of Cardiac Surgery, Medical University of Innsbruck, Innsbruck, Austria; b Department of Cardiovascular Surgery, University Heart Center Freiburg-Bad Krozingen, University Medical Center Freiburg, Freiburg, Germany; c Cardio-Thoracic Surgery Department, Maastricht University Medical Centre, Maastricht, the Netherlands; d University Clinic of Internal Medicine III, Cardiology and Angiology, Medical University of Innsbruck, Austria; e Department of Cardiothoracic Surgery, Uppsala University Hospital, Uppsala, Sweden; f Wallenberg Centre for Molecular and Translational Medicine, Institute of Medicine, University of Gothenburg, Sweden; g Department of Molecular and Clinical Medicine, Institute of Medicine, University of Gothenburg, Sweden; h Department of Laboratory Medicine, Sahlgrenska Academy, University of Gothenburg, Gothenburg, Sweden; i Department of Cardiothoracic Surgery, Landspitali University Hospital, Faculty of Medicine, University of Iceland, Reykjavik, Iceland; j Central Institute of Clinical Chemistry and Laboratory Medicine, Medical University of Innsbruck, Innsbruck, Austria; k Department of Cardiology and Angiologie, University Heart Center Freiburg-Bad Krozingen, University Medical Center Freiburg, Freiburg, Germany; l Department of Cardiothoracic Surgery, Sahlgrenska University Hospital, Gothenburg, Sweden; m Department of Molecular and Clinical Medicine, Institute of Medicine, Sahlgrenska Academy, University of Gothenburg, Gothenburg, Sweden; n Institute of Clinical Epidemiology, Public Health, Health Economics, Medical Statistics and Informatics, Medical University of Innsbruck, Innsbruck, Austria; o Ignaz Semmelweis Institute, Interuniversity Institute for Infection Research, Medical University of Vienna, Vienna, Austria; p Department of Public Health and Primary Care, University of Cambridge, Cambridge, United Kingdom

**Keywords:** CABG, cardiac biomarker, cardiac surgery, myocardial injury, periprocedural myocardial infarction

## Abstract

**Background:**

Accurate diagnosis of perioperative myocardial infarction after coronary artery bypass grafting remains challenging due to uncertainty over postoperative cardiac biomarker interpretation.

**Objectives:**

The authors aimed to evaluate the prognostic value and performance of postoperative creatine kinase–myocardial band (CK-MB) and high-sensitivity cardiac troponin T (hs-cTnT) and explore a combined approach to identify patients at risk of early death.

**Methods:**

This multicenter cohort study involved 9,856 patients undergoing isolated coronary artery bypass grafting at hospitals in Innsbruck, Freiburg, Gothenburg, and Reykjavik. A combined biomarker matrix was created to assess synergistic effects. Associations of biomarkers with 30-day all-cause mortality were evaluated using multivariable Cox regression (adjusted HR) and area under the receiver-operating curve (AUC) analysis.

**Results:**

Thirty-day all-cause mortality was 2.0%. Median peak values within 72 hours postsurgery were 2.24 (Q1-Q3: 1.66-3.36) × upper reference limit for CK-MB and 52.06 (Q1-Q3: 29.79-90.00) × upper reference limit for hs-cTnT. Both postoperative CK-MB (adjusted HR: 1.95; 95% CI: 1.79-2.12; *P* < 0.001) and hs-cTnT (adjusted HR: 1.71; 95% CI: 1.61-1.82; *P* < 0.001) were associated with higher mortality risk per doubling in concentration independent of EuroSCORE II. Predictive accuracy of CK-MB and hs-cTnT was similar (AUC: 0.787 vs 0.784; *P* = 0.99), yet stratification using Youden-derived thresholds identified distinct patient subgroups with limited overlap. In a combined analysis, mortality risk was increased only when CK-MB was in the top tertile and hs-cTnT in the mid (adjusted HR: 4.34; 95% CI: 1.78-10.62; *P* < 0.001) or top tertile (adjusted HR: 14.78; 95% CI: 6.85-31.88; *P* < 0.001).

**Conclusions:**

The combined use of CK-MB and hs-cTnT enables a simple and robust risk-stratification strategy for early postoperative mortality, with direct implications for clinical surveillance and future cardiac surgery trials.

Myocardial injury and infarction remain among the most clinically relevant complications of coronary artery bypass grafting (CABG), contributing significantly to early postoperative mortality and impaired long-term outcomes.^1^ Timely identification of patients at risk is therefore essential to guide postoperative care and to initiate timely re-revascularization if necessary.^2^ Moreover, perioperative myocardial infarction (PMI) remains a key endpoint in landmark trials comparing CABG and percutaneous coronary intervention (PCI).^3^ Yet, the diagnosis of PMI continues to pose challenges. Routine postoperative biomarker elevations frequently arise from surgical manipulation and ischemia-reperfusion injury, making it sometimes difficult to distinguish benign biomarker release from clinically meaningful myocardial damage.^4^

Current definitions of PMI, including the Fourth Universal Definition of Myocardial Infarction, recommend the use of cardiac troponins and require biomarker elevation in conjunction with ischemic signs such as new electrocardiogram changes or regional wall motion abnormalities.^5^ In parallel, consensus statements have proposed isolated biomarker thresholds to define myocardial injury after cardiac surgery in the absence of additional ischemic evidence.^6^ These efforts aim to provide objective criteria for identifying clinically relevant injury, yet threshold recommendations remain largely empirical, and only a limited number of studies have been validated against hard outcomes such as mortality.^7^, ^8^, ^9^, ^10^

High-sensitivity cardiac troponins (hs-cTn) are now widely used for diagnosing acute coronary syndromes due to their excellent sensitivity and cardiac specificity.^11^ Conversely, creatine kinase–myocardial band (CK-MB), once the cornerstone of myocardial infarction diagnosis, has been largely phased out in cardiology practice.^12^ Nonetheless, CK-MB remains in widespread use in cardiac surgery world-wide and is still included in current PMI definitions due to its ability to detect procedural myocardial injury. Importantly, studies that have rigorously compared hs-cTn and CK-MB in the context of cardiac surgery are scarce, and none have assessed whether a combined biomarker strategy could improve clinical risk stratification or facilitate a more individualized approach to PMI diagnosis.

Therefore, we initiated this multicenter cohort study to address this knowledge gap. By using a contemporary population of nearly 10,000 patients undergoing isolated CABG across 4 tertiary centers, we evaluated the prognostic utility of high-sensitivity cardiac troponin T (hs-cTnT) and CK-MB with respect to 30-day all-cause mortality. Both CK-MB and hs-cTnT were routinely measured and available in all patients.

Our study pursued 3 main objectives:

1. to evaluate the prognostic association between postoperative CK-MB and hs-cTnT concentrations and 30-day all-cause mortality after isolated CABG;
2. to compare directly the predictive accuracy of CK-MB and hs-cTnT for early postoperative risk stratification; and
3. to determine whether a combined biomarker strategy—integrating information captured by CK-MB and hs-cTnT—offers superior discrimination of high-risk patients and enables reliable rule-out of increased early mortality risk.

## Methods

### Study population and data collection

This multicenter cohort study included 10,297 consecutive patients who underwent elective isolated CABG at 4 tertiary cardiac surgery centers: Innsbruck (Austria), Freiburg (Germany), Reykjavík (Iceland), and Gothenburg (Sweden) between 2010 and 2023. Patients without data on survival (n = 325) were excluded from the analysis (Supplemental Figure 1). hs-cTnT and CK-MB were assessed preoperatively and serially during the first 72 hours postoperatively in all patients, in accordance with institutional protocols. Patients without postoperative biomarker data (n = 116) were excluded from the analysis (Supplemental Figure 1). Center-specific sampling schedules were as follows: Innsbruck: samples were collected at hospital admission and postoperatively at 4 hours, 12 hours, 24 hours, 48 hours, and 72 hours. Freiburg: samples were collected at hospital admission and postoperatively at 0 hour, 4 hours, 12 hours, and 24 hours. An additional 2-hour sample was collected if there was clinical suspicion of myocardial hypoperfusion. Reykjavik and Gothenburg: samples were collected immediately preoperatively, 3 hours postoperatively, on the morning after surgery, and on postoperative days 2, 3, and 4. For each patient, the peak value recorded within this 72-hour window was used for statistical analysis. For hs-cTnT, all centers used the Elecsys Troponin T high-sensitive assay (Roche Diagnostics). For CK-MB, the following assays were applied: Innsbruck and Freiburg—Cobas CK-MB assay (Roche Diagnostics); Reykjavík—Elecsys CK-MB (Roche Diagnostics); and Gothenburg—Abbott Alinity STAT CK-MB (Abbott Laboratories). Survival data were obtained by national federal statistical institutes and patient health record databases. The median follow-up for fatal outcomes was 73 months (Q1-Q3: 32-119). Follow-up for all-cause mortality within the 30-day postoperative period was complete in all patients. This study was performed in accordance with the Declaration of Helsinki, and permission to use anonymized data without patient consent for this study was obtained from the Institutional Review Boards (Innsbruck Medical University IRB number 1009/2024; Swedish Ethical Review Authority 2023-00298-02; University of Freiburg 25-1208-S1-retro) Reykjavik (National Bioethics Committee of Iceland 10-009-V18).

### Study endpoints and statistical analysis

The primary endpoint of this study was 30-day all-cause mortality. In addition, the prognostic value of postoperative CK-MB and hs-cTnT levels for 30-day mortality was evaluated and compared. Categorical variables are presented as counts and percentages. Continuous variables were assessed for normality using the Kolmogorov-Smirnov test. Normally distributed data are expressed as mean ± SD and compared using the Student’s *t*-test, and non-normally distributed data as median with 25th-75th percentiles (Q1-Q3) and compared using the Mann-Whitney *U* test. For the comparison of nominal data, the chi-square test or Fisher exact test was applied. The percentages shown in the manuscript are of the non-missing data of each parameter. Percentages might therefore not match in regard to the total cohort size. Cardiac biomarkers (CK-MB and hs-cTnT) were normalized to the respective 99th-percentile upper reference limit (URL). For hs-cTnT, a uniform URL of 14 ng/L was applied across all centers; for CK-MB, the URLs were 25 U/L in Innsbruck and Freiburg, 7 U/L in Reykjavík, and sex-specific in Gothenburg (3.4 U/L in women, 5.1 U/L in men). The association between peak postoperative cardiac biomarker levels and 30-day mortality was assessed using area under the receiver-operating characteristic curve (AUC) analysis based on logistic regression analysis. Optimal cutpoint values for CK-MB and hs-cTnT were determined by Youden's J statistic to maximize sensitivity and specificity. For the 2 biomarkers, the AUCs were compared using DeLong's test. Cox proportional hazards regression was used to estimate HRs for mortality associated with biomarker concentrations above the identified cutoffs. To evaluate the continuous relationship between biomarker levels and mortality, HRs were also calculated per doubling of biomarker values. Patients were further stratified into tertiles based on CK-MB and hs-cTnT concentrations and cross-classified into 9 groups for interaction analysis. All regression models were adjusted for EuroSCORE II (reflecting preoperative risk and comorbidities) and study center. HRs are reported with 95% CIs. A 2-sided *P* value of <0.05 was considered statistically significant. Statistical analyses were conducted using IBM SPSS Statistics version 24 (IBM Corp), GraphPad Prism Version 9.4.1 (GraphPad Software), and R statistical software (version 4.4.3, R Core Team, 2024).

## Results

### Study population and surgical characteristics

Of the included patients, 1,778 (18.0%) were female; the median age was 68.0 years (Q1-Q3: 61.0–74.0) (Table 1). A history of prior myocardial infarction was present in 33.8% of patients, and 23.8% had undergone PCI before surgery. The median preoperative left ventricular ejection fraction was 57% (Q1-Q3: 50.0–60.0). Most patients presented with NYHA functional class II (39.9%) or III (35.9%) symptoms. Preoperative median serum creatinine was 0.99 mg/dL (Q1-Q3: 0.85–1.15), and 1.2% of patients were on chronic dialysis. The median EuroSCORE II was 1.50% (Q1-Q3: 0.95–2.59). The median aortic cross-clamp and cardiopulmonary bypass times were 65 (Q1-Q3: 49-83) and 97 (Q1-Q3: 78-121) minutes, respectively. Postoperatively, 79 patients (0.8%) required perioperative extracorporeal membrane oxygenation, and 372 (4.0%) underwent temporary hemofiltration. The 30-day all-cause mortality rate was 2.0% (n = 194) (Tables 1 and 2).

**Table 1:** Baseline Characteristics of the Study Population Stratified by Postoperative Biomarker Levels

	All Patients (N = 9,856)	Below Both Thresholds (n = 5,732)	Above hs-cTnT Threshold Only (n = 732)	Above CK-MB Threshold Only (n = 2,051)	Above Both Thresholds (n = 1,341)
Preoperative characteristics					
Female	1,778 (18.0%)	943 (16.5%)	76 (10.3%)	455 (22.2%)	304 (22.7%)
Age (y)	68 (61, 74)	67 (61, 74)	68 (61, 74)	69 (61, 75)	70 (63, 76)
BMI (kg/m^2^)	27.4 (25.0, 30.2)	27.3 (25.0, 30.1)	27.0 (25.0, 30.0)	27.8 (25.1, 31.0)	27.2 (24.8, 30.0)
Diabetes	4,966 (52.3%)	2,630 (47.6%)	249 (35.4%)	1,385 (71.3%)	702 (54.3%)
Dyslipidemia	7,157 (73.2%)	4,410 (77.5%)	638 (87.9%)	1,155 (56.7%)	954 (71.7%)
Hypertension	7,912 (80.2%)	4,686 (81.7%)	656 (89.6%)	1,473 (71.8%)	1,097 (81.8%)
History of smoking	4,717 (49.8%)	2,673 (48.5%)	336 (47.8%)	1,058 (53.6%)	650 (50.4%)
Creatinine (mg/dL)	0.99 (0.85, 1.15)	0.97 (0.84, 1.11)	1.00 (0.88, 1.22)	1.00 (0.87, 1.22)	1.00 (0.88, 1.22)
On dialysis	105 (1.2%)	52 (1.0%)	23 (4.0%)	13 (0.6%)	17 (1.4%)
COPD	856 (8.7%)	478 (8.4%)	61 (8.3%)	192 (9.4%)	125 (9.4%)
Stroke	399 (5.9%)	246 (5.5%)	43 (6.1%)	43 (5.6%)	67 (7.7%)
History of MI	3,335 (33.8%)	1,900 (33.1%)	353 (48.2%)	521 (25.4%)	561 (41.8%)
History of PCI	1,720 (23.8%)	945 (22.5%)	158 (29.4%)	389 (25.8%)	228 (23.1%)
LVEF (%)	57 (50, 60)	58 (50, 60)	59 (50, 64)	55 (50, 60)	55 (49, 60)
NYHA functional class					
I	1,261 (15.8%)	780 (14.5%)	86 (15.2%)	251 (16.2%)	144 (12,3%)
II	3,215 (39.9%)	2,067 (44.5%)	230 (38.8%)	545 (32.8%)	373 (34.4%)
III	2,889 (35.9%)	1,604 (34.6%)	227 (38.3%)	602 (36.2%)	456 (42.0%)
IV	686 (8.5%)	287 (6.1%)	36 (6.2%)	201 (13%)	162 (14%)

Values are median (25th, 75th percentiles) or n (%). The table presents baseline characteristics for all patients undergoing isolated CABG (n = 9,856), stratified by biomarker status according to postoperative CK-MB and hs-cTnT levels relative to their respective Youden-derived thresholds (100.96 ×URL for hs-cTnT) and 2.79 ×URL for CK-MB. Patients were grouped as follows: below both thresholds (n = 5,732), above hs-cTnT threshold only (N = 732), above CK-MB threshold only (n = 2,051), and above both thresholds (n = 1,341).

BMI = body mass index; CABG = coronary artery bypass grafting; CK-MB = creatine kinase–myocardial band; COPD = chronic obstructive pulmonary disease; hs-cTnT = high-sensitivity cardiac troponin T; LVEF = left ventricular ejection fraction; MI = myocardial infarction; PCI = percutaneous coronary intervention; URL = upper reference limit.

**Table 2:** Operative and Postoperative Characteristics Stratified by Postoperative Biomarker Levels

	All Patients (N = 9,856)	Below Both Thresholds (n = 5,732)	Above hs-cTnT Threshold Only (n = 732)	Above CK-MB Threshold Only (n = 2,051)	Above Both Thresholds (n = 1,341)
Operative characteristics					
Reoperation	312 (3.4%)	158 (3.0%)	21 (3.5%)	43 (2.1%)	90 (7.3%)
EuroSCORE II	1.50 (0.95, 2.59)	1.38 (0.90, 2.26)	1.50 (0.92, 2.78)	1.59 (1.00, 2.73)	2.14 (1.20, 4.33)
Cross clamp time (min)	65 (49, 83)	62 (48, 76)	73 (59, 88)	65 (47, 90)	77 (59, 100)
Prefusion time (min)	97 (78, 121)	90 (74, 110)	111 (93, 135)	101 (81, 128)	120 (93, 154)
Postoperative characteristics					
ECMO	79 (0.8%)	15 (0.3%)	8 (1.2%)	3 (0.1%)	53 (4.1%)
Ultrafiltration	372 (4.0%)	134 (2.4%)	59 (8.8%)	41 (2.1%)	138 (11%)
ICU stay duration (d)	1.00 (0.00, 1.12)	1.00 (0.00, 1.00)	0.00 (0.00, 2.00)	1.00 (1.00, 1.00)	2.00 (1.00, 5.00)
30-d mortality	194 (2.0%)	31 (0.5%)	8 (1.1%)	35 (1.7%)	120 (8.9%)

Values are median (25th, 75th percentiles) or n (%). This table summarizes intraoperative and early postoperative parameters in the overall cohort (n = 9,856) and across biomarker-defined subgroups based on postoperative CK-MB and hs-cTnT levels. Groups include patients below both biomarker thresholds (n = 5,732), above the hs-cTnT threshold only (n = 732), above the CK-MB threshold only (n = 2,051), and above both thresholds (n = 1,341). EuroSCORE II reflects predicted perioperative mortality risk. ICU stay duration is recorded in days.

ECMO = extracorporeal membrane oxygenation; ICU = intensive care unit; other abbreviations as in Table 1.

### Troponin and CK-MB: complementary markers for 30-day postoperative mortality

Peak biomarker values within 72 hours after surgery were 52.06 (Q1-Q3: 29.79–90.00) × the URL for hs-cTnT and 2.24 (Q1-Q3: 1.66–3.36) × URL for CK-MB (Figure 1A). The receiver-operating characteristic curve analysis demonstrated that postoperative biomarker elevation performed well at distinguishing between patients who died within 30 days of CABG and patients who survived. Both hs-cTnT and CK-MB showed comparable AUCs (hs-cTnT: 0.784; CK-MB: 0.787; both *P* < 0.001). The difference in predictive accuracy between the 2 biomarkers was not statistically significant (AUC difference = 0.003; *P* = 0.86) (Figure 1B). Based on Youden’s J statistic, the cutoff values providing the best balance between sensitivity and specificity were 100.96 × URL for hs-cTnT (sensitivity 0.660, specificity 0.799) and 2.79 × URL for CK-MB (sensitivity 0.799, specificity 0.665). These thresholds were used to stratify patients into groups (Tables 1 and 2). Elevated postoperative biomarker levels were associated with increased risk of 30-day all-cause mortality in an unadjusted model but also when adjusted for EuroSCORE II (Table 3). For hs-cTnT, each doubling in concentration conferred a 71% increase in mortality risk (adjusted HR: 1.71; 95% CI: 1.61-1.82; *P* < 0.001), while the corresponding increase for CK-MB was 95% (adjusted HR: 1.95; 95% CI: 1.79-2.12; *P* < 0.001). When categorizing patients into tertiles based on postoperative biomarker concentration, hs-cTnT showed a gradual and consistent increase in risk across all levels. Compared to patients in the lowest hs-cTnT tertile, the adjusted HR was 2.15 (95% CI: 1.19-3.89; *P* = 0.011) in the mid group and 7.39 (4.39-12.45, *P* < 0.001) in the top tertile group (Table 3). In contrast, elevated risk for CK-MB was only confined to the top tertile group (middle: adjusted HR: 1.33; 95% CI: 0.72-2.46; *P* = 0.36; top: adjusted HR: 7.28; 95% CI: 4.24-11.92; *P* < 0.001). To translate these findings into clinically actionable thresholds, we next identified optimal cutoff values for both biomarkers.

**Figure 1:**
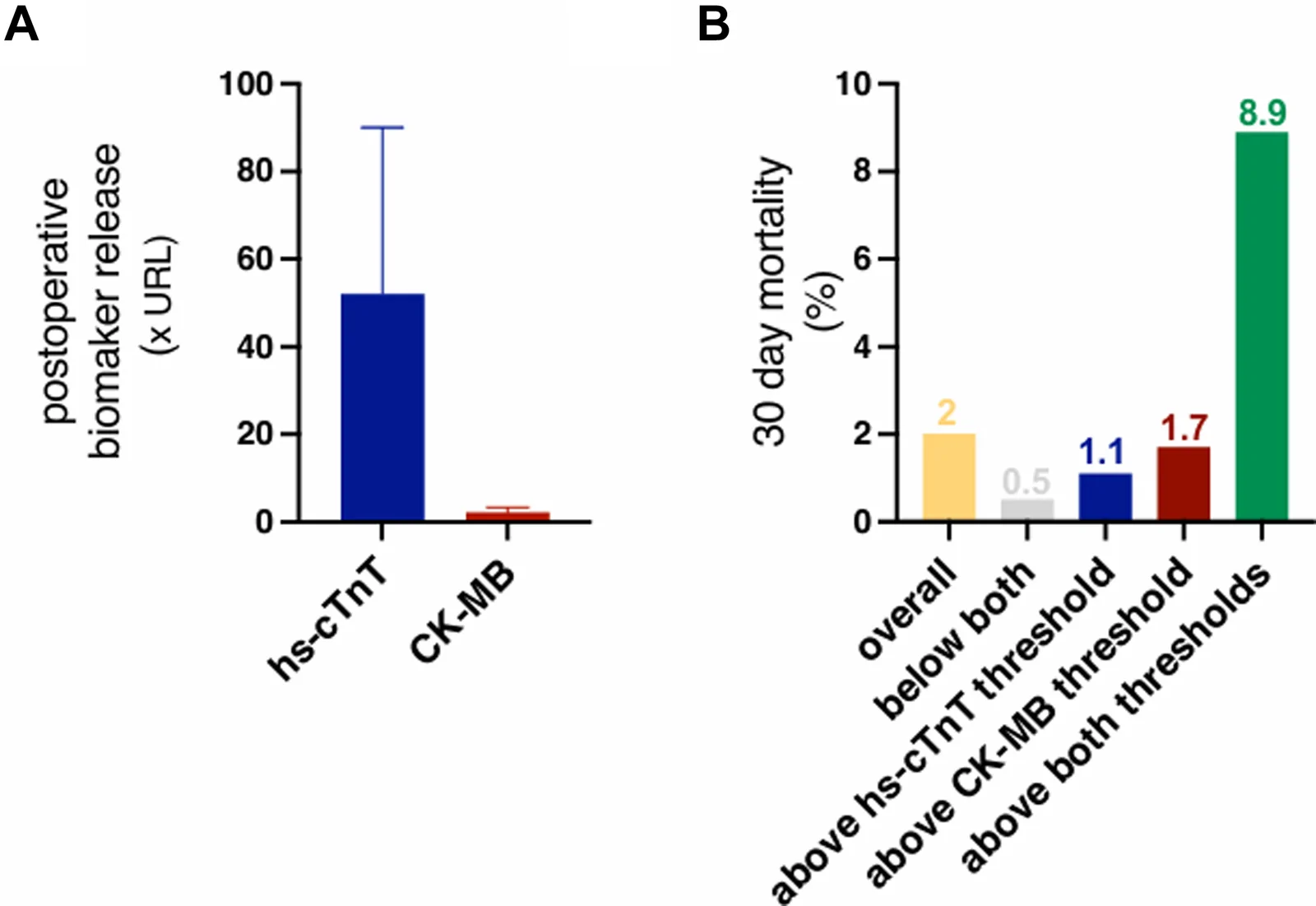
Postoperative Biomarker Levels and Predictive Accuracy for 30-Day Mortality After Coronary Artery Bypass Grafting (A) Distribution of postoperative hs-cTnT and CK-MB levels expressed as multiples of the 99th percentile upper reference limit (xURL) following CABG. (B) 30-day mortality rates across biomarker-defined subgroups. CABG = coronary artery bypass grafting; CK-MB = creatine kinase–myocardial band; hs-cTnT = high-sensitivity cardiac troponin T; URL = upper reference limit.

**Table 3:** Association of Postoperative Biomarker Levels With 30-Day Mortality

	Bottom Third	Middle Third	Top Third	HR (95% CI) per Twice the Level
hs-cTnT				
No. of patients	3,300	3,272	3,284	
No. of events	16	36	142	
% 30-day mortality	0.5%	1.1%	4.3%	
Unadjusted model				
HR (95% CI)	Ref*.*	2.28 (1.27-4.11)	9.11 (5.43-15.27)	
*P* value	Ref*.*	0.006	<0.001	
Adjusted for ES II				
HR (95% CI)	Ref*.*	2.15 (1.19-3.88)	7.39 (4.39-12.45)	1.71 (1.61-1.82)
*P* value	Ref*.*	0.011	<0.001	<0.001
CK-MB				
No. of patients	3,334	3,285	3,237	
No. of events	18	25	151	
% 30-day mortality	0.5%	0.8%	4.7%	
Unadjusted model				
HR (95% CI)	Ref*.*	1.41 (0.77-2.59)	8.82 (5.54-14.38)	
*P* value	Ref*.*	0.27	<0.001	
Adjusted for ES II				
HR (95% CI)	Ref*.*	1.33 (0.72-2.46)	7.28 (4.24-11.92)	1.95 (1.79-2.12)
*P* value	Ref*.*	0.36	<0.001	<0.001

Patients were stratified into tertiles according to postoperative hs-cTnT and CK-MB levels, expressed as multiples of the upper reference limit. The table shows the number of patients, number of events, and 30-day mortality rates within each tertile. HRs with 95% CIs and corresponding *P* values were derived from Cox proportional hazards models. Results are presented for both unadjusted and EuroSCORE II–adjusted models. Additionally, HRs per doubling of biomarker level are shown to reflect the continuous prognostic effect.

Both unadjusted and adjusted HRs were presented.

Abbreviations as in Table 1.

### Threshold-based risk stratification using postoperative hs-cTnT and CK-MB levels

Figure 2 compares patient risk stratification when using guideline-recommended cutoffs, together with the data-driven Youden’s J-based cutoff values for both markers. Guideline thresholds varied widely in prevalence and discriminatory power, identifying between 1.6% and 98.7% of patients, with corresponding mortality rates ranging from 2.0% to 25.5%. The Youden-derived thresholds identified intermediate-sized subgroups (21.0% for hs-cTnT, 34.3% for CK-MB) that carried significantly increased mortality risk (HRs >6, both *P* < 0.001) (Figure 1C, Table 2). Further tertile analyses showed that hs-cTnT was associated with a gradual, dose-dependent increase in risk across tertiles, while CK-MB only displayed elevated risk confined to the top third (Table 3). The 2 biomarkers identified overlapping but not identical patient populations, with 21% of the cohort exceeding the hs-cTnT threshold, 34.4% the CK-MB threshold, and 13.6% exceeding both (Figure 1C). Thirty-day mortality was 0.5% in patients below both thresholds, 1.1% with isolated hs-cTnT elevation, 1.7% with isolated CK-MB elevation, and 8.9% when both biomarkers were elevated (Figure 1D). This observation prompted a combined biomarker analysis to assess whether dual-marker profiling could improve both high-risk identification and low-risk rule-out.

**Figure 2:**
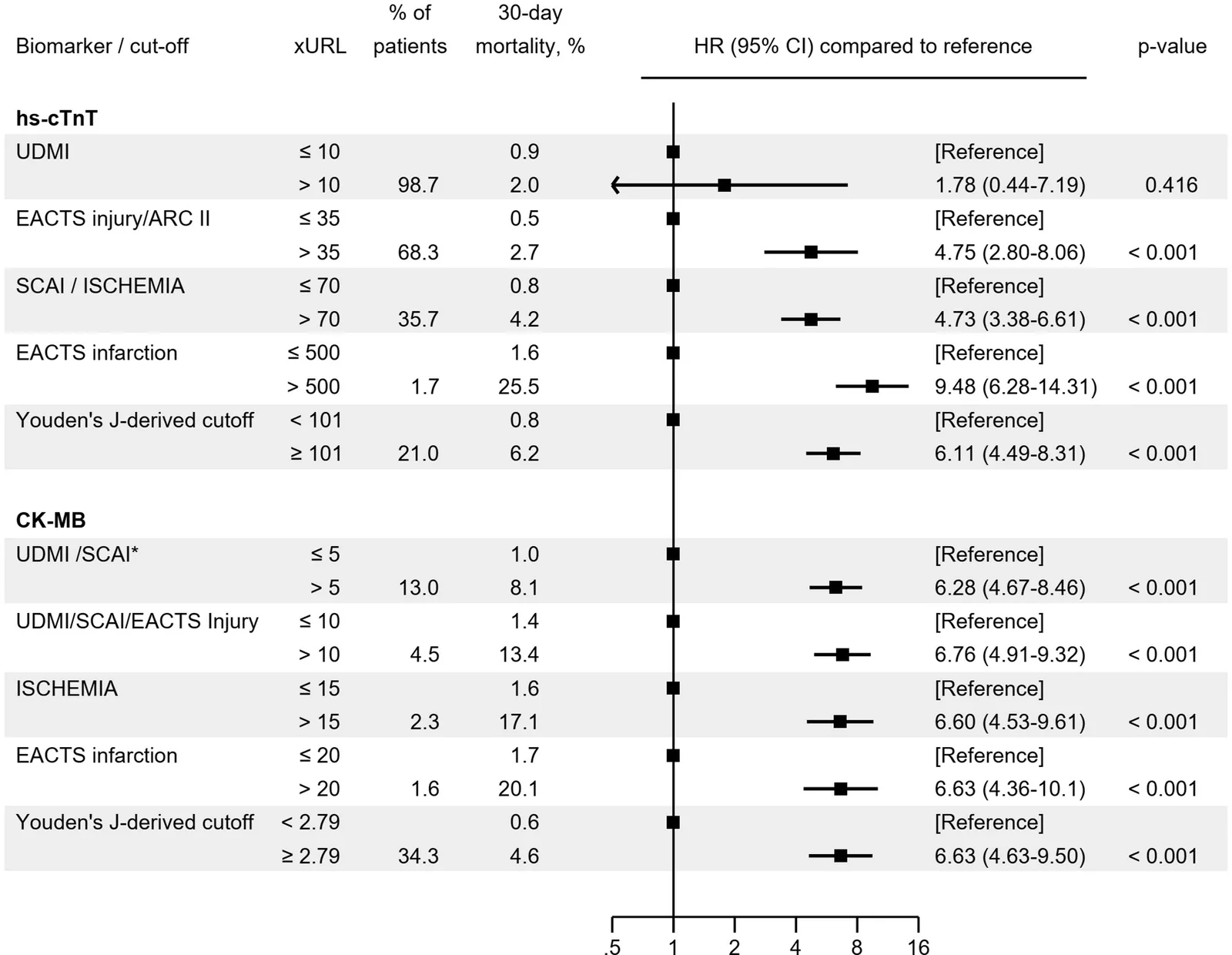
Association of Biomarker Thresholds With 30-Day Mortality After Coronary Artery Bypass Grafting The figure compares guideline-recommended and Youden-derived thresholds for hs-cTnT and CK-MB in relation to 30-day all-cause mortality. For each threshold, the percentage of patients above the cutoff, observed mortality, HR, and *P* value are reported. Models were adjusted for EuroSCORE II. Results illustrate marked variability in threshold prevalence and prognostic impact, highlighting the added value of data-driven cutoffs for postoperative risk stratification. EACTS = European Association for Cardio-Thoracic Surgery; SCAI = Society for Cardiovascular Angiography and Interventions; UDMI = Universal Definition of Myocardial Infarction; other abbreviations as in Figure 1.

### Combined biomarker profiling for early risk-stratification

To test if the combination of both markers would improve risk stratification, patients were cross-classified by hs-cTnT and CK-MB tertiles, resulting in a 3×3 matrix (Table 4, Supplemental Tables 2 and 3). This combined stratification revealed that a significantly increased risk of 30-day mortality occurred only when CK-MB levels were in the top tertile (>2.84 × URL) and hs-cTnT levels were either in the mid (9.3%, adjusted HR: 4.34; 95% CI: 1.78-10.17; *P* = 0.001) or top tertile (17.2%, adjusted HR: 14.78; 95% CI: 6.85-31.88; *P* < 0.001; likelihood ratio test: *P* = 0.003). All other biomarker combinations, including isolated elevations in either marker, were not associated with significantly elevated risk.

**Table 4:** Combined Prognostic Impact of CK-MB and hs-cTnT Tertiles on 30-Day Mortality

hs-cTnT	CK-MB		
	Bottom Third ≤1.8 × URL	Middle Third 1.8-2.8	Top Third >2.8 × URL
Bottom third ≤36.4 × URL	n = 1,640 (16.6%) died within 30 days: 7 (0.4%) Reference	n = 1,033 (10.5%) died within 30 days: 4 (0.4%) HR: 0.90 (95% CI: 0.26-3.07) *P* = 0.86	n = 627 (6.4%) died within 30 days: 5 (0.8%) HR: 2.02 (95% CI: 0.63-6.48) *P* = 0.24
Middle third 36.4-73.6 × URL	n = 1,191 (12.1%) died within 30 days: 8 (0.7%) HR; 1.63 (95% CI: 0.59-4.55) *P* = 0.35	n = 1,164 (11.8%) died within 30 days: 11 (0.9%) HR: 1.99 (95% CI: 0.76-5.24) *P* = 0.16	n = 917 (9.3%) died within 30 days: 17 (1.9%) HR: 4.34 (95% CI: 1.78-10.62) *P* = 0.001
Top third >73.6 × URL	n = 503 (5.1%) died within 30 days: 3 (0.6%) HR; 1.4 (95% CI: 0.36-5.54) *P* = 0.62	n = 1,088 (11.0%) died within 30 days: 10 (0.9%) HR: 2.19 (95% CI: 0.82-5.84) *P* = 0.12	n = 1,693 (17.2%) died within 30 days: 129 (7.6%) HR: 14.78 (95% CI: 6.85-31.88) *P* < 0.001

Patients were stratified into 9 groups based on cross-classification of postoperative CK-MB and hs-cTnT tertiles, defined by multiples of the upper reference limit (URL). The group size is presented as number of patients and percentage of the total cohort, together with the number of patients died within 30 days and the corresponding 30-day mortality rate within each group. The reference group comprised patients in the lowest tertile for both biomarkers (CK-MB ≤1.84 × URL and hs-cTnT ≤36.43 × URL). HRs with 95% CIs were calculated for 30-day mortality using Cox regression, adjusted for EuroSCORE II and study center. Results highlight the incremental prognostic value of combined biomarker elevation.

Abbreviations as in Table 1.

### Biomarker thresholds for predictive accuracy across sensitivity analyses

To assess the robustness of our findings, we performed multiple sensitivity analyses (Supplemental Table 1). The associations between elevated postoperative biomarker levels and 30-day mortality remained consistent across all models. When adjusting for center effects, HRs for mortality were 7.92 (95% CI: 5.45-11.51) for patients above the CK-MB threshold, 6.71 (95% CI: 4.91-9.18) for those above the hs-cTnT threshold, and 8.42 (95% CI: 6.22-11.40) for patients exceeding both thresholds (all *P* < 0.001). Similar results were observed in analyses restricted to elective procedures and to patients with normal renal function (glomerular filtration rate ≥50 mL/min/1.73 m^2^), with the latter group showing an even stronger association for combined biomarker elevation (HR: 11.82; 95% CI: 7.98-17.49; *P* < 0.001).

## Discussion

In this large European contemporary cohort of patients undergoing CABG, we investigated the prognostic value of 2 routinely measured postoperative cardiac biomarkers: CK-MB and hs-cTnT—with respect to 30-day all-cause mortality. We found that elevations in both biomarkers were independently and significantly associated with early postoperative mortality, with similar overall discriminatory performance. Although hs-cTnT and CK-MB thresholds showed comparable prognostic accuracy, they importantly identified largely distinct subsets of at-risk patients. Most notably, a novel dual-marker approach, combining both hs-cTnT and CK-MB tertiles, allowed refined both stratification and identified a subgroup with markedly increased risk of early death, which otherwise would not have been captured by either biomarker alone. These findings underscore the complementary nature of the 2 biomarkers and support their combined use for early risk stratification following cardiac surgery (Central Illustration).

**Central Illustration:**
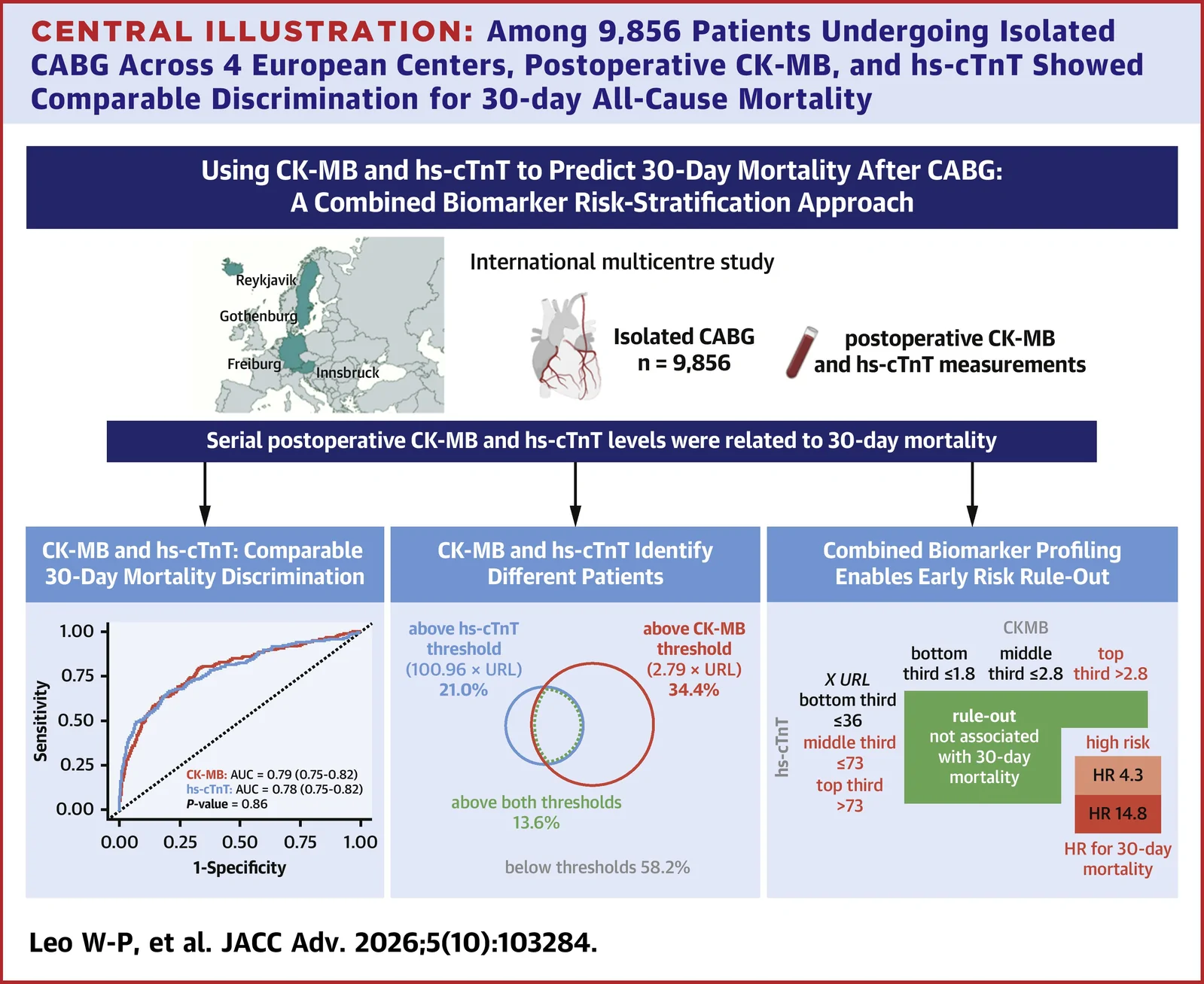
Among 9,856 Patients Undergoing Isolated CABG Across 4 European Centers, Postoperative CK-MB, and hs-cTnT Showed Comparable Discrimination for 30-day All-Cause Mortality Despite similar overall prognostic performance, Youden-derived thresholds identified partly distinct patient groups. Combined biomarker profiling demonstrated that elevated hs-cTnT was associated with increased mortality only in the presence of top-tertile CK-MB elevation, whereas patients with CK-MB in the lower or middle tertiles were not at increased 30-day mortality risk. These findings support a combined CK-MB/hs-cTnT rule-out approach for early postoperative mortality risk stratification. Abbreviations as in Figure 1.

Cardiac biomarker elevations are strong predictors of adverse outcomes following cardiac surgery, regardless of the procedure performed.^8^ The VISION study, including over 13,000 patients across multiple centers, showed that postoperative high-sensitivity cardiac troponin I concentrations were independently associated with 30-day mortality, with clinically relevant thresholds exceeding 200× the URL in patients undergoing isolated CABG or open cardiac valve procedures.^13^ In a separate analysis of over 5,000 patients from randomized trials, elevations of CK-MB above 2.5× URL were associated with a significantly increased risk of long-term mortality, with a near-linear relationship between CK-MB concentration and 5-year death rates.^14^ While these studies established the independent prognostic value of troponin and CK-MB, they did not assess the potential clinical utility of combining both biomarkers. The current study addresses this limitation by demonstrating that a dual-marker approach significantly improves early risk stratification and more reliably enables rule-out of 30-day mortality after CABG.

Biologically, hs-cTnT and CK-MB may reflect partly distinct but overlapping aspects of myocardial injury. High-sensitivity troponins are capable of detecting even subtle cardiomyocyte injury; however, the mechanisms underlying troponin release remain debated and may include reversible membrane alterations, apoptosis, cardiomyocyte turnover, and microscopic necrosis rather than purely reversible injury. In contrast, CK-MB has traditionally been considered more closely associated with more pronounced procedural myocardial injury, including ischemic injury and focal necrosis.^4^ Interestingly, in our cohort, hs-cTnT displayed a stepwise association with 30-day mortality across tertiles, suggesting dose-response behavior, while CK-MB exhibited a more discrete threshold effect. This observation may indicate that hs-cTnT and CK-MB capture different dimensions of perioperative myocardial injury, although this interpretation remains hypothesis-generating and requires further mechanistic validation. These findings support the continued utility of CK-MB in the postoperative setting, despite its declining role in general clinical cardiology.^15^

Another important observation was that the 2 biomarkers identified only partially overlapping at-risk populations. This divergence suggests that they may capture different injury patterns or pathophysiological mechanisms. CK-MB is less affected by noncardiac factors such as renal dysfunction and may offer superior specificity in patients with chronic kidney disease or in women, who typically exhibit lower baseline troponin concentrations and altered biomarker kinetics.^16^ Conversely, hs-cTnT may be more sensitive for detecting diffuse or subclinical myocardial injury, particularly in younger or lower-risk patients. These differences support the rationale for using individualized biomarker strategies postoperatively after CABG, wherein patient characteristics such as sex, renal function, or prior cardiac history can affect the choice of biomarker to rely on and the used cutoff values. Future research should aim to develop integrated clinical algorithms or calculators incorporating these parameters to enhance risk prediction in cardiac surgery.^17^

The most important finding and novelty of our study lies in the dual-marker stratification framework. By cross-classification of patients into 9 groups based on biomarker tertiles, we could identify a small but high-risk subgroup with CK-MB in the top tertile and hs-cTnT in the mid or top tertiles, who exhibited a markedly increased risk of 30-day mortality (HR up to 14.8). In contrast, and importantly, isolated elevations in either biomarker alone were not significantly associated with excess risk. This synergistic effect has not been previously reported and suggests that dual-marker profiling substantially enhances risk discrimination. Equally important, patients with both biomarkers below the derived thresholds had a favorable short-term prognosis, underscoring the potential of this approach for reliable early risk stratification. Collectively, these findings support the use of dual-marker profiling as a simple and clinically actionable tool to guide postoperative monitoring and resource allocation.

These results carry important clinical and research implications. Existing definitions of PMI, such as the Fourth Universal Definition of MI, primarily rely on troponin thresholds but also acknowledge the absence of surgery-specific criteria.^5^ Similarly, a recent position paper by the European Association for Cardio-Thoracic Surgery emphasized the need for more precise and evidence-based definitions of myocardial injury following CABG.^6^ Our findings directly address this knowledge gap by proposing a data-driven, implementable strategy that enhances diagnostic precision and enables early identification of both high- and low-risk patients.

Beyond improving individual patient management, our study may be a further platform for the design and interpretation of future clinical trials in cardiac surgery. As PMI is frequently used as a component of composite endpoints in studies evaluating revascularization strategies or cardioprotective interventions, the lack of standardized, surgery-specific biomarker thresholds contributes to endpoint heterogeneity and limits comparability across trials. By demonstrating that a combined hs-cTnT and CK-MB strategy improves early mortality risk stratification and identifies clinically meaningful subgroups, our results support the incorporation of dual-marker frameworks into future trial protocols. Such approaches could enhance endpoint definition, improve risk adjustment, and ultimately strengthen the methodological rigor and interpretability of outcomes in cardiac research, both regarding PCI and myocardial revascularization.

## Strengths and limitations

Several methodological strengths support the validity of our findings. These include the use of a well-characterized surgical cohort, routine biomarker assessment within a standardized time window, and multivariable adjustment for key clinical confounders. In addition, the multicenter design and large sample size of our cohort provide a degree of statistical robustness. To further support this, we performed a sensitivity analysis adjusted for participating centers (Supplemental Table 1), which confirmed that our observations were independent of center effects. Nonetheless, certain limitations must be acknowledged. Our analysis focused on peak biomarker concentrations within 72 hours postoperatively; therefore, we believe that future studies should incorporate biomarker trajectories or time-to-peak values for further providing additional granularity. The sampling schedules varied between centers and therefore present a limitation. Moreover, we assessed the primary outcome with 30-day all-cause mortality, only showing association but not proving causality. Moreover, patients with missing data regarding the 30-day endpoint were removed rather than censored, potentially leading to selection bias. Unfortunately, the data set did not retain the serial postoperative biomarker measurements or their corresponding sampling time points, but only the peak value per patient. Therefore, the timing of the postoperative enzyme peak cannot be reconstructed, even approximately within center-specific sampling schedules. This precludes analysis of early vs late peak elevation and may limit mechanistic interpretation of biomarker release patterns, as well as the assessment of potential differences in release and clearance kinetics between hs-cTnT and CK-MB. Furthermore, the association between biomarker release and long-term prognosis of the patients remains to be determined. Another limitation relates to the definition of biomarker thresholds. We used Youden’s J to derive the proposed thresholds, which represents a balanced statistical compromise by identifying the cutoff with the highest combined sensitivity and specificity. However, this approach does not necessarily represent the optimal threshold for every clinical purpose. In particular, if the intended clinical use is to rule out early, thresholds prioritizing high sensitivity and negative predictive value may be more appropriate. Conversely, very low thresholds may lead to overdiagnosis and limited clinical manageability, whereas very high thresholds may risk underdiagnosis of patients at increased risk. Thus, the proposed thresholds should be interpreted as data-derived values for risk stratification rather than definitive clinical decision cutoffs. Prospective validation in even larger multicenter cohorts in Europe and in non-European centers worldwide should incorporate biomarker dynamics and could further enhance the clinical utility of this approach.

## Conclusions

By systematically comparing 2 widely used cardiac biomarkers and introducing a novel dual-marker risk model, this study provides both immediate clinical implications for postoperative risk-stratification and broader academic relevance. In a subgroup of patients, a robust biomarker-based stratification strategy may improve the diagnostic accuracy for 30-day mortality risk. Further studies based on this approach could inform future PMI definitions and strengthen the validity of PMI as an endpoint in clinical trials. In the context of increasing emphasis on individualized medicine and standardized outcome reporting, our findings may contribute to refining future definitions of PMI and enhance benchmarking of surgical outcomes.

Perspectives **COMPETENCY IN MEDICAL KNOWLEDGE:** Postoperative creatine kinase–myocardial band and high-sensitivity cardiac troponin T provide complementary prognostic information after isolated coronary artery bypass grafting. Both biomarkers are associated with 30-day all-cause mortality, independently of preoperative risk. Combined assessment identifies distinct risk groups, with the highest mortality risk among patients with marked elevations of both biomarkers and low observed mortality among those below both study-derived thresholds.

**TRANSLATIONAL OUTLOOK:** Prospective external validation is needed to establish clinically applicable thresholds and determine whether biomarker-guided surveillance and management improve outcomes. Future studies should incorporate standardized sampling, biomarker trajectories, and clinical evidence of ischemia to clarify how combined biomarker assessment could inform perioperative myocardial infarction definitions and trial endpoints. The proposed thresholds should be considered exploratory risk-stratification tools rather than established diagnostic or treatment criteria.

## Funding support and author disclosures

Dr Peter has received consultancy fees from Novartis Pharmaceuticals. Dr Anders has received consultancy fees from Pharmacosmos and LFB Biotechnologies outside the present work. Dr Martin is a consultant for Terumo Aortic, Endospan, and Medira, and a shareholder of TEVAR Ltd and Ascense Medical. All other authors have reported that they have no relationships relevant to the contents of this paper to disclose.
