# Supplementary material for: Using CK-MB and hs-cTnT to Predict 30-Day Mortality After CABG: A Combined Biomarker Risk-Stratification Approach
Source: JACC Adv. 2026 Sep 25;5(10):103284. doi: 10.1016/j.jacadv.2026.103284 (PMC13635570; doi:10.1016/j.jacadv.2026.103284)
Supplement: Supplementary file 1 [file mmc1.docx]

Supplemental Material

Supplemental Figure 1: Flow chart

Flow diagram depicting patient inclusion for the analysis. A total of 10,297 patients undergoing isolated coronary artery bypass grafting (CABG) between 2010 and 2020 at four international cardiac centers were screened. After exclusion of 441 patients due to missing survival or biomarker data, 9,856 patients were included in the final analysis. Reasons for exclusion and center-specific enrollment numbers are detailed.

Abbreviations: CABG = coronary artery bypass grafting

| 30-day mortality | | | |
| --- | --- | --- | --- |
|  | above CK-MB Threshold | above hs-cTnT Threshold | above both Thresholds |
|  | 6.63 (4.63-19.50) | 6.11 (4.49-8.31) | 8.55 (6.32-11.58) |
|  | <0.001 | <0.001 | <0.001 |
| Adjusted for center |  |  |  |
| HR (95% CI) | 7.92 (5.45-11.51) | 6.71 (4.91-9.18) | 8.42 (6.22-11.40) |
| p-value | <0.001 | <0.001 | <0.001 |
| elective cases only |  |  |  |
| HR (95% CI) | 7.81 (4.71-12.98) | 6.23 (4.08-9.51) | 9.76 (6.42-14.84) |
| p-value | <0.001 | <0.001 | <0.001 |
| Normal kidney function only (GFR >= 50 mL/min/1.73 m²) | | |  |
| HR (95% CI) | 8.85 (5.50-14.28) | 8.46 (5.65-12.67) | 11.82 (7.98-17.49) |
| p-value | <0.001 | <0.001 | <0.001 |
| model adjusted for the EuroSCORE II | | | |

**Supplemental Table 1:** Sensitivity Analyses for the Association of Biomarker Thresholds with 30-Day Mortality

Hazard ratios (HRs) with 95% confidence intervals (CI) for 30-day mortality are presented for patients above the CK-MB threshold, above the hs-cTnT threshold, and above both thresholds. All models are adjusted for EuroSCORE II. Sensitivity analyses include models additionally adjusted for study center, limited to elective cases only, and restricted to patients with preserved renal function (glomerular filtration rate [GFR] ≥ 50 mL/min/1.73 m²). All associations remained statistically significant across subgroups.

Abbreviations: CI = confidence interval; CK-MB = creatine kinase–myocardial band; GFR = glomerular filtration rate; HR = hazard ratio; hs-cTnT = high-sensitivity cardiac troponin T.

**Supplemental Table 2:** Baseline Characteristics of the Study Population Stratified by hs-cTnT Levels

|  |  | Postoperative hs-cTnT | | |  |
| --- | --- | --- | --- | --- | --- |
| Characteristic | **Overall** N = 9,856*^1^* | **Lower third** N = 3,300*^1^* | middle third N = 3,272*^1^* | **Upper third** N = 3,284*^1^* | p-value*^2^* |
| Sex (female) | 1,778 (18%) | 642 (19%) | 544 (17%) | 592 (18%) | 0.012 |
| Age (years) | 68.0 (61.0, 74.0) | 67.0 (60.0, 73.0) | 68.0 (61.0, 74.5) | 69.0 (61.8, 75.0) | <0.001 |
| Body mass index | 27.4 (25.0, 30.2) | 27.7 (25.1, 30.9) | 27.2 (25.0, 30.1) | 27.1 (24.9, 30.0) | <0.001 |
| History of Hypertension | 7,912 (80%) | 2,479 (75%) | 2,653 (81%) | 2,780 (85%) | <0.001 |
| Diabetes Mellitus | 4,966 (52%) | 1,877 (60%) | 1,615 (51%) | 1,474 (46%) | <0.001 |
| Insulin dependent Diabetes Mellitus | 761 (10%) | 205 (8.8%) | 272 (11%) | 284 (11%) | 0.036 |
| Dyslipidemia | 7,157 (73%) | 2,154 (66%) | 2,439 (75%) | 2,564 (79%) | <0.001 |
| Peripheral artery disease | 894 (12%) | 255 (11%) | 307 (12%) | 332 (13%) | 0.009 |
| On dialysis | 105 (1.2%) | 13 (0.4%) | 34 (1.2%) | 58 (2.1%) | <0.001 |
| History of COPD | 856 (8.7%) | 271 (8.2%) | 306 (9.4%) | 279 (8.5%) | 0.2 |
| History of smoking | 4,717 (50%) | 1,620 (51%) | 1,535 (49%) | 1,562 (49%) | 0.084 |
| History of stroke | 399 (5.9%) | 103 (5.5%) | 131 (5.5%) | 165 (6.5%) | 0.3 |
| Cerebrovascular disease | 321 (12%) | 28 (9.3%) | 109 (11%) | 184 (13%) | 0.054 |
| History of myocardial infarction | 3,335 (34%) | 889 (27%) | 1,057 (32%) | 1,389 (42%) | <0.001 |
| Previous PCI | 1,720 (24%) | 565 (24%) | 534 (22%) | 621 (25%) | 0.081 |
| NYHA |  |  |  |  | <0.001 |
| 0 | 229 (2.8%) | 119 (4.5%) | 70 (2.6%) | 40 (1.5%) |  |
| 1 | 1,032 (13%) | 356 (13%) | 328 (12%) | 348 (13%) |  |
| 2 | 3,215 (40%) | 1,070 (40%) | 1,128 (42%) | 1,017 (37%) |  |
| 3 | 2,889 (36%) | 878 (33%) | 953 (36%) | 1,058 (39%) |  |
| 4 | 686 (8.5%) | 242 (9.1%) | 186 (7.0%) | 258 (9.5%) |  |
| Preoperative creatinine (mg/dl) | 0.99 (0.85, 1.15) | 0.98 (0.84, 1.12) | 0.98 (0.85, 1.14) | 1.00 (0.87, 1.20) | <0.001 |
| EuroScoreII (%) | 1.50 (0.95, 2.59) | 1.35 (0.91, 2.20) | 1.49 (0.95, 2.50) | 1.70 (1.03, 3.28) | <0.001 |
| Urgency of surgery |  |  |  |  | <0.001 |
| Elective | 6,395 (68%) | 2,175 (67%) | 2,177 (70%) | 2,043 (66%) |  |
| Emergency/Salvage | 574 (6.1%) | 95 (2.9%) | 129 (4.1%) | 350 (11%) |  |
| Urgent | 2,494 (26%) | 994 (30%) | 820 (26%) | 680 (22%) |  |
| Cross-clamp time (min) | 65.00 (49.00, 83.00) | 56.00 (42.00, 71.00) | 66.00 (51.00, 83.00) | 73.00 (57.00, 92.00) | <0.001 |
| Cardio-pulmonary bypass time (min) | 97.00 (78.00, 121.00) | 83.00 (68.00, 101.00) | 98.00 (82.00, 120.00) | 112.00 (90.00, 139.00) | <0.001 |
| ECMO | 79 (0.8%) | 0 (0%) | 9 (0.3%) | 70 (2.3%) | <0.001 |
| Days in the ICU | 1.00 (0.00, 1.12) | 1.00 (1.00, 1.00) | 1.00 (0.00, 1.00) | 1.00 (0.00, 3.00) | <0.001 |
| Postoperative hemofiltration | 372 (4.0%) | 37 (1.1%) | 86 (2.8%) | 249 (8.3%) | <0.001 |
| Postoperative hs-cTnT (x URL) | 52.06 (29.79, 90.00) | 23.29 (16.86, 29.86) | 52.14 (43.86, 61.43) | 114.93 (90.00, 166.36) | <0.001 |
| Postoperative CK-MB (x URL) | 2.24 (1.66, 3.36) | 1.86 (1.40, 2.56) | 2.12 (1.64, 3.04) | 2.92 (2.12, 5.20) | <0.001 |
| Died after 30 days | 194 (2.0%) | 16 (0.5%) | 36 (1.1%) | 142 (4.3%) | <0.001 |
| *^1^*n (%); Median (Q1, Q3) | | | | | |
| *^2^*Pearson's Chi-squared test; Kruskal-Wallis rank sum test | | | | | |

**Supplemental Table 3:** Baseline Characteristics of the Study Population Stratified by CK-MB Levels

|  |  | Postoperative CK-MB | | |  |
| --- | --- | --- | --- | --- | --- |
| Characteristic | **Overall** N = 9,856*^1^* | **Lower third** N = 3,334*^1^* | middle third N = 3,285*^1^* | **Upper third** N = 3,237*^1^* | p-value*^2^* |
| Sex (female) | 1,778 (18%) | 479 (14%) | 566 (17%) | 733 (23%) | <0.001 |
| Age (years) | 68.00 (61.00, 74.00) | 67.00 (60.00, 73.00) | 68.00 (61.00, 74.00) | 69.00 (62.00, 75.00) | <0.001 |
| Body mass index | 27.40 (25.00, 30.20) | 27.10 (25.00, 30.00) | 27.40 (25.00, 30.12) | 27.68 (25.00, 30.61) | 0.002 |
| History of Hypertension | 7,912 (80%) | 2,777 (83%) | 2,687 (82%) | 2,448 (76%) | <0.001 |
| Diabetes Mellitus | 4,966 (52%) | 1,386 (43%) | 1,565 (49%) | 2,015 (66%) | <0.001 |
| Insulin dependent Diabetes Mellitus | 761 (10%) | 259 (8.9%) | 297 (11%) | 205 (11%) | 0.019 |
| Dyslipidemia | 7,157 (73%) | 2,673 (81%) | 2,487 (76%) | 1,997 (62%) | <0.001 |
| Peripheral artery disease | 894 (12%) | 347 (12%) | 321 (12%) | 226 (12%) | 0.8 |
| On dialysis | 105 (1.2%) | 50 (1.7%) | 29 (1.0%) | 26 (0.8%) | 0.004 |
| History of COPD | 856 (8.7%) | 259 (7.8%) | 300 (9.1%) | 297 (9.2%) | 0.071 |
| History of smoking | 4,717 (50%) | 1,517 (47%) | 1,549 (49%) | 1,651 (53%) | <0.001 |
| History of stroke | 399 (5.9%) | 157 (5.6%) | 141 (5.7%) | 101 (6.7%) | 0.3 |
| Cerebrovascular disease | 321 (12%) | 140 (11%) | 118 (12%) | 63 (14%) | 0.5 |
| History of myocardial infarction | 3,335 (34%) | 1,147 (35%) | 1,154 (35%) | 1,034 (32%) | 0.018 |
| Previous PCI | 1,720 (24%) | 552 (24%) | 572 (25%) | 596 (22%) | 0.073 |
| NYHA |  |  |  |  | <0.001 |
| 0 | 229 (2.8%) | 51 (1.9%) | 70 (2.6%) | 108 (4.1%) |  |
| 1 | 1,032 (13%) | 400 (15%) | 356 (13%) | 276 (11%) |  |
| 2 | 3,215 (40%) | 1,263 (46%) | 1,083 (40%) | 869 (33%) |  |
| 3 | 2,889 (36%) | 864 (32%) | 1,016 (37%) | 1,009 (39%) |  |
| 4 | 686 (8.5%) | 144 (5.3%) | 191 (7.0%) | 351 (13%) |  |
| Preoperative creatinine (mg/dl) | 0.99 (0.85, 1.15) | 0.98 (0.84, 1.12) | 0.98 (0.84, 1.12) | 1.00 (0.87, 1.22) | <0.001 |
| EuroScoreII (%) | 1.50 (0.95, 2.59) | 1.30 (0.87, 2.14) | 1.48 (0.98, 2.49) | 1.77 (1.08, 3.33) | <0.001 |
| Urgency of surgery |  |  |  |  | <0.001 |
| Elective | 6,395 (68%) | 2,320 (73%) | 2,143 (69%) | 1,932 (61%) |  |
| Emergency/Salvage | 574 (6.1%) | 115 (3.6%) | 145 (4.7%) | 314 (9.9%) |  |
| Urgent | 2,494 (26%) | 754 (24%) | 826 (27%) | 914 (29%) |  |
| Cross-clamp time (min) | 65.00 (49.00, 83.00) | 60.00 (47.00, 74.00) | 66.00 (52.00, 82.00) | 70.00 (51.00, 94.00) | <0.001 |
| Cardio-pulmonary bypass time (min) | 97.00 (78.00, 121.00) | 88.00 (71.00, 108.00) | 97.00 (81.00, 119.00) | 109.00 (85.00, 138.00) | <0.001 |
| ECMO | 79 (0.8%) | 9 (0.3%) | 15 (0.5%) | 55 (1.7%) | <0.001 |
| Days in the ICU | 1.00 (0.00, 1.12) | 1.00 (0.00, 1.00) | 1.00 (0.00, 1.00) | 1.00 (1.00, 2.00) | <0.001 |
| Postoperative hemofiltration | 372 (4.0%) | 101 (3.2%) | 97 (3.1%) | 174 (5.8%) | <0.001 |
| Postoperative hs-cTnT (x URL) | 52.06 (29.79, 90.00) | 37.06 (21.57, 58.49) | 54.06 (31.43, 86.36) | 77.86 (42.14, 143.57) | <0.001 |
| Postoperative CK-MB (x URL) | 2.24 (1.66, 3.36) | 1.48 (1.24, 1.68) | 2.24 (2.04, 2.52) | 4.37 (3.40, 6.59) | <0.001 |
| Died after 30 days | 194 (2.0%) | 18 (0.5%) | 25 (0.8%) | 151 (4.7%) | <0.001 |
| *^1^*n (%); Median (Q1, Q3) | | | | | |
| *^2^*Pearson's Chi-squared test; Kruskal-Wallis rank sum test | | | | | |
